# Penis trauma after fish bites: a case report

**DOI:** 10.11604/pamj.2025.51.93.48445

**Published:** 2025-08-13

**Authors:** Amrizal Amrizal, Thia Prameswarie, Dientyah Nur Anggina, Miranti Dwi Hartanti, Raden Ayu Tanzila, Indri Ramayanti

**Affiliations:** 1 Department of Surgery, Faculty of Medicine, University of Muhammadiyah Palembang, Sumatra, Indonesia,; 2 Department of Parasitology, Faculty of Medicine, University of Muhammadiyah Palembang, Sumatra, Indonesia,; 3 Department of Public Health, Faculty of Medicine, University of Muhammadiyah Palembang, Sumatra, Indonesia,; 4 Department of Pharmacology, Faculty of Medicine, University of Muhammadiyah Palembang, Sumatra, Indonesia,; 5 Department of Physiology, Faculty of Medicine, University of Muhammadiyah Palembang, Sumatra, Indonesia

**Keywords:** Penile injury, fish bite, meatoplasty, case report

## Abstract

Penile trauma is challenging because it impacts both physical and mental well-being. It is uncommon because the penis is well-protected by its position. We report the case of an 11-year-old boy who presented with penile glans partial amputation and total transection of the urethra resulting from a fish bite injury while swimming in the river. The patient received emergency care, including wound irrigation, anti-tetanus shots, broad-spectrum antibiotics, pain medication, and pre-op preparation for surgical debridement. Meatoplasty for the conservation of the penile glans was performed. The patient was given antibiotics for five days and a urinary catheter for 14 days. Suture removal was done on the 21^st^ day after surgery. After three months, the patient was free of urinary problems, and there was no stricture in the new meatus.

## Introduction

Penile trauma can be caused by traffic accidents, animal bites, burns, injuries due to a pants zipper, or during the circumcision process [1]. Trauma from frequent animal bites in children can be both dog bites and mammalian animals [2]. Many types of animals can cause bite wounds on male external genitalia, including dogs, snakes, goats, horses, and donkeys. Cases of trauma from dog bites are 3.2 times more prevalent in children than in adults [3]. The severity of penile trauma can range from minor to severe injuries, and in some cases, even total amputation [4]. Trauma from fish bites is rarely reported. We report the case of an 11-year-old boy who underwent successful metoplasty for the conservation of the penile gland.

## Patient and observation

We report a case of an 11-year-old male who presented following traumatic genital injury with penile glans partial amputation and total transection of the urethra resulting from a fish bite injury while swimming in the river. The patient had successful meatoplasty for the conservation of the penile gland.

**Patient information:** a boy, 11 years old, brought by his parents to the emergency unit of AR Bunda Hospital with the chief complaint of a fish bite on the head of the penis about 14 hours ago. The patient has a history of swimming in the river without pants.

**Figure 1:**
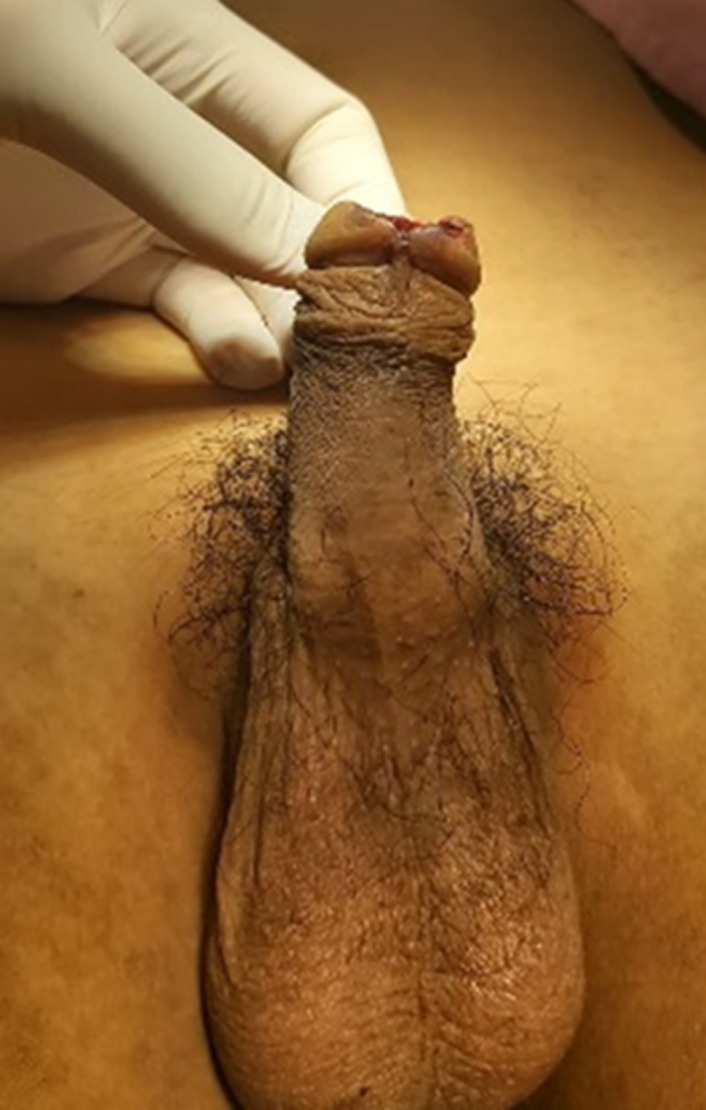
penis following fish bite injury, visible ventral distal trauma of penile grade 3

**Figure 2:**
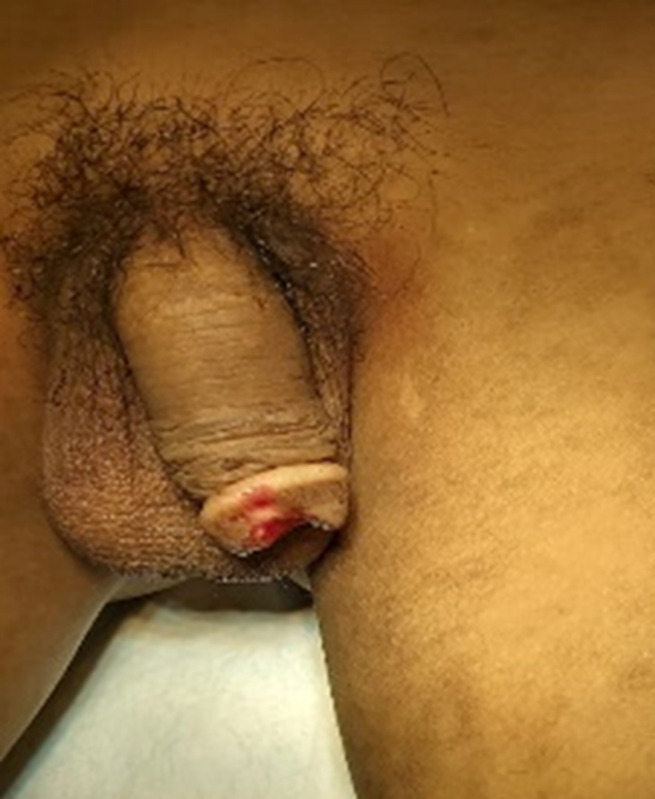
penis following fish bite injury, Dorsal side of penile distal trauma grade 3

**Figure 3:**
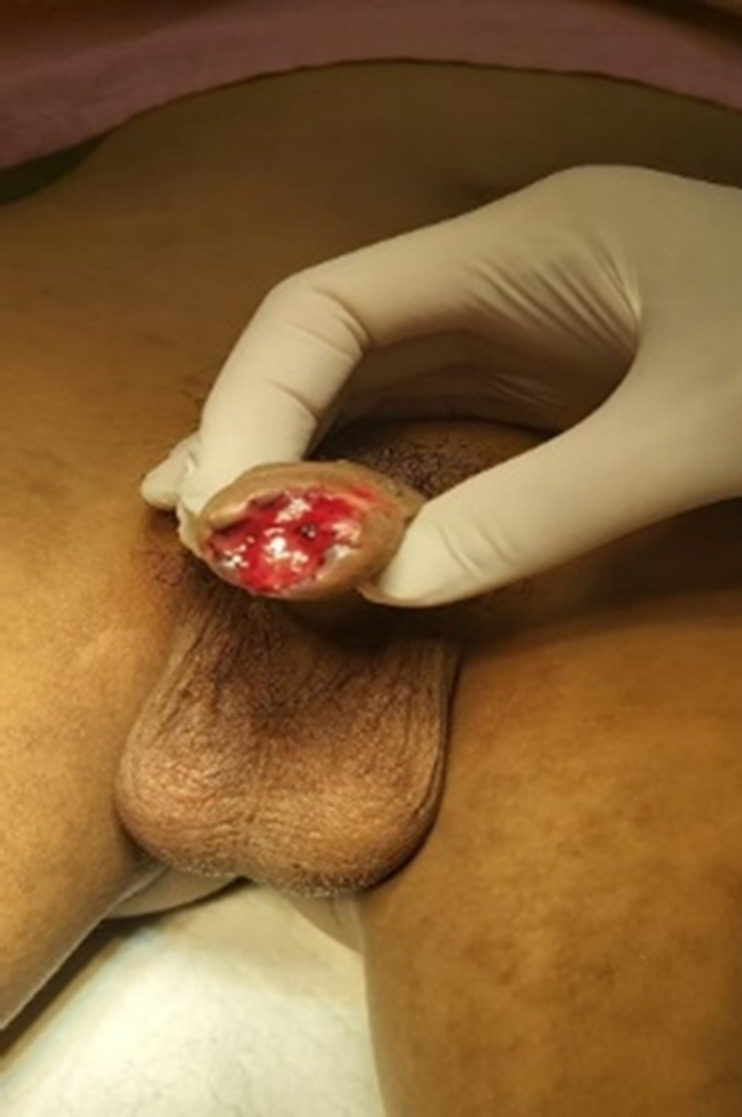
traumatic meatus urethra

**Clinical findings:** in physical examination, there was penile gland amputation up to 0.5 cm from the corona glandis, irregular wound edges, total transection of the urethra, and no source of active bleeding (Figure 1, Figure 2, Figure 3). Initial examination of the patient was compos mentis and vital signs were within normal limits. The patient was diagnosed as grade 3 penile trauma based on the American Association for the Surgery of Trauma (AAST) classification.

**Diagnostic assessment:** the following tests were requested: a complete blood count (CBC), blood typing, a clotting profile, a urea test, and a serum creatinine test. The results of the biological investigations were as follows: hemoglobin of 12g/dL and a white blood cell count of 11.200 cells/mm^3^. The clotting profile and renal function tests were normal.

**Figure 4:**
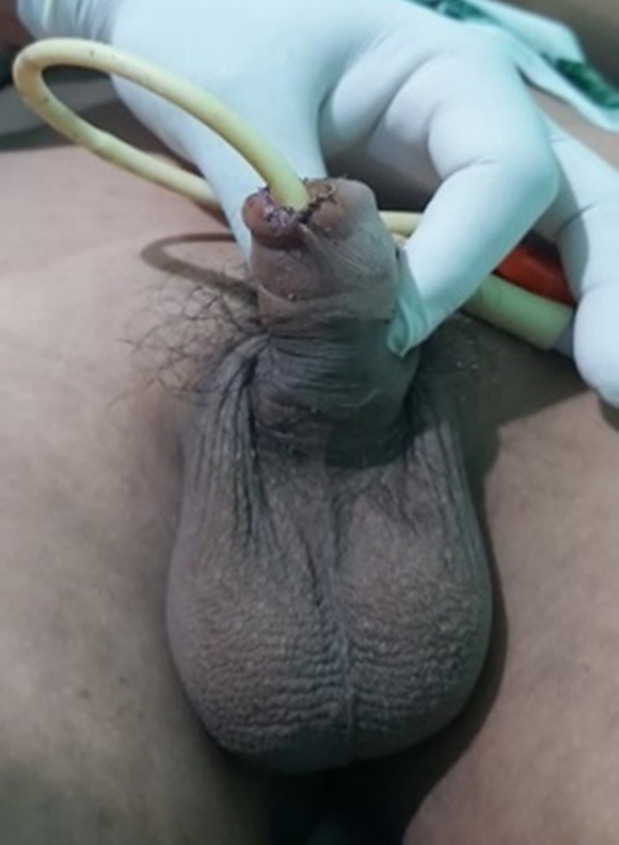
ventral view post-meatoplasty

**Therapeutic intervention:** following the primary and secondary surveys, emergency management treatment was wound irrigation, anti-tetanus serum, empiric broad-spectrum antibiotics, analgesics medication, and prepared for surgical debridement. Patients receive treatment in the form of performed meatoplasty for the conservation of the penile glans. The patient entered the operating room, and general anesthesia for debridement, penile glans revision as a partial amputation of the glans penis and proximal segment of urethra was identified. Foley catheter No. 14 was inserted, and meatoplasty was performed using VICRYL© 4-0 interrupted spongiourethral suture (Figure 4). The Foley catheter is maintained for 2 weeks.

**Figure 5:**
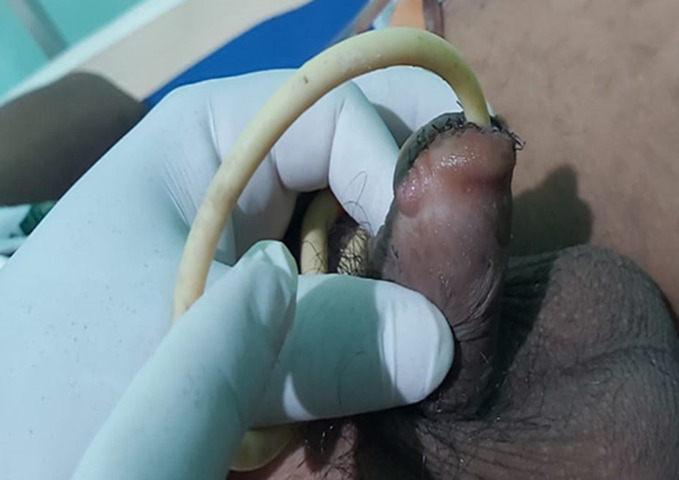
lateral view post meatoplasty, fourteenth day after surgery

**Figure 6:**
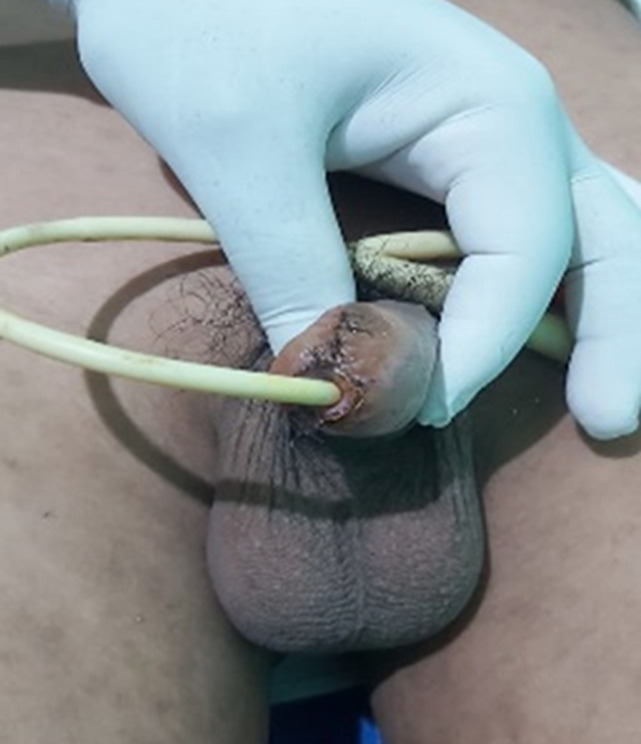
front view post meatoplasty, fourteenth day after surgery

**Follow-up and outcome:** the patient was given antibiotics for 5 days and a urinary catheter was placed for 14 days (Figure 5, Figure 6). Suture removal was carried out on the 21^st^ day after surgery. After 3 months, the patient did not complain of urinary disturbances, and no stricture was found in the “new” meatus.

**Patient's perspective:** the patient was satisfied with the medical attention and follow-up. He received excellent care from the time he arrived at the emergency department until after he was discharged. He was pleased that the medical team had treated his penis quickly.

**Informed consent:** consent was obtained from the patient for publication of this article.

## Discussion

Penile trauma is caused by several etiologies; these differences in causes can lead to different types of traumas, ranging from mild to severe. Penile open trauma is a rare case in the field of urology; penile trauma in children can have psychological, physical, and emotional effects on patients and their parents [5,6].

Based on a report from Rashid *et al*. genital trauma in men is classified by penis anatomical location, divided into type I, namely trauma that includes the distal penis with its proximal part still remaining. Type II includes the loss of the corpora penis while still leaving the crus of the penis. Type III is perineal urethrostomy, which requires urethral catheterization. Type IV is a trauma urinary diversion that requires a suprapubic catheter [7]. According to the AAST, penile trauma is classified based on severity into five degrees. Degree 1 is cutaneous laceration/contusion. Second degree is Buck's fascia (cavernosum) laceration without tissue loss. Grade 3 is cutaneous avulsion or laceration through the glans or meatus with a cavernosal or urethral defect of less than 2 cm. Grade 4 is partial penectomy with a cavernosal or urethral defect greater than 2 cm. Degree 5 is total penectomy [8].

Penile trauma that occurs in this case belongs to type 1, trauma at the tip of the penis/urethral meatus is often followed by complications of narrowing the meatus urethra (meatal stenosis), which will result in complaints of pain when urinating, difficulty urinating, urinary tract infections, and kidney function damage. To prevent such complications in these patients, meatoplasty or surgical procedures are performed to dilate the urethral estuary. Meatoplasty is one of the treatments for meatal stenosis that requires general anaesthesia in theatre. Meatoplasty has a better prognosis than others [9]. Management of penile trauma due to animal bites must also pay attention to the importance of preventing tetanus, rabies, and infection of the wound. Bite wounds have a great risk of infection if not treated within 48 hours after the trauma. Microorganisms can come from the mouth of the biter or from the environment. In addition, the genital area is also a high-risk area for infection [10].

## Conclusion

Cases of penile trauma caused by fish bites are rarely reported. The management given to these patients is in the form of debridement, administration of anti-tetanus serum, and antibiotics. The surgery performed is meatoplasty. Meatoplasty is a curative surgical procedure to prevent narrowing/stenosis of the urethra due to trauma to the distal penile.

## References

[1] Djordjevic ML, Bumbasirevic MZ, Krstic Z, Bizic MR, Stojanovic BZ, Miocinovic R (2014). Severe penile injuries in children and adolescents: reconstruction modalities and outcomes. Urology.

[2] Youness C, Yassine D, Alafifi M, Moataz A, Mohamed D, Adil D (2021). Dog bite to the external genitals: About six cases. Annal Cas Rep Rev.

[3] Bertozzi M, Appignani A (2013). The management of dog bite injuries of genitalia in paediatric age. Afr J Paediatr Surg.

[4] Perovic SV, Djinovic RP, Bumbasirevic MZ, Santucci RA, Djordjevic ML, Kourbatov D (2009). Severe penile injuries: a problem of severity and reconstruction. BJU Int.

[5] Boumas N, Mougougou A, Ndang Ngou-Milama S, Angue Nguema M, Massande J (2022). Traumatic penile partial amputation of unusual occurrence: A case report. Urol Case Rep.

[6] Oranusi CK, Nwofor A (2014). Traumatic penile injuries: Mechanisms and problems of treatment in a tertiary institution in Nigeria. Niger J Clin Pract.

[7] Rashid M, Sarwar SU (2005). Avulsion injuries of the male external genitalia: classification and reconstruction with the customised radial forearm free flap. Br J Plast Surg.

[8] American Association for the Surgery of Trauma (2023). Injury Scoring Scale: A Resource for Trauma Care Professional. Injury Scoring Scale - The American Association for the Surgery of Trauma.

[9] Francis GI, Victor A (2022). Management of Meatal Stenosis in Port Harcourt: A Ten-Year Retrospective Study. Open Journal of Urology.

[10] Abrahamian FM, Goldstein EJ (2011). Microbiology of animal bite wound infection. Clin Microbiol Rev.

